# Nongenomic Mechanisms of PTEN Regulation

**DOI:** 10.1155/2012/379685

**Published:** 2012-03-25

**Authors:** Jimmie E. Fata, Shawon Debnath, Edmund C. Jenkins, Marcia V. Fournier

**Affiliations:** ^1^Department of Biology, College of Staten Island, 2800 Victory Boulevard, Staten Island, NY 10314, USA; ^2^Biology Doctoral Program, City University of New York Graduate Center, 365 Fifth Avenue, New York, NY 10016, USA; ^3^Biochemistry Doctoral Program, City University of New York Graduate Center, 365 Fifth Avenue, New York, NY 10016, USA; ^4^BIOARRAY Therapeutics Inc., Venture Development Center, UMASS, 100 Morrissey Boulevard, Boston, MA 02125, USA

## Abstract

A large amount of data supports the view that PTEN is a bona fide tumor suppressor gene. However, recent evidence suggests that derailment of cellular localization and expression levels of functional nonmutated PTEN is a determining force in inducing abnormal cellular and tissue outcomes. As the cellular mechanisms that regulate normal PTEN enzymatic activity resolve, it is evident that deregulation of these mechanisms can alter cellular processes and tissue architecture and ultimately lead to oncogenic transformation. Here we discuss PTEN ubiquitination, PTEN complex formation with components of the adherens junction, PTEN nuclear localization, and microRNA regulation of PTEN as essential regulatory mechanisms that determine PTEN function independent of gene mutations and epigenetic events.

## 1. PTEN: A Unique Dual-Specificity Phosphatase

PTEN (phosphatase and tensin homolog deleted on chromosome ten)/MMAC (mutated in multiple advanced cancers) has been identified simultaneously by two research groups as a candidate tumor suppressor gene located at 10q23 and encoding 403 amino acids [1, 2]. Another group identified the same gene in the search for new dual-specific phosphatases and named it TEP-1 (TGF-*β* regulated and epithelial cell-enriched phosphatase) [3]. PTEN is one of the most common targets of mutation in human cancer, with a mutation frequency approaching that of the tumor suppressor gene p53, and it is also mutated in inherited cancer predisposition disorders. PTEN belongs to the protein tyrosine phosphatase family with phosphatase activity on both lipids and proteins. PTEN's lipid phosphatase catalyzes the conversion of phosphatidylinositol-(3,4,5)-triphosphate (PIP_3_) to phosphatidylinositol-4,5-bisphosphate (PIP_2_) [4, 5] and plays an important role in the PI3K pathway by catalyzing degradation of PIP_3_ generated by PI3K. This inhibits PI3K downstream targets, mainly PKB-Akt [6–10]. It should be noted, however, that lipid phosphatase attenuated or inactive PTEN mutants have been reported to still retain some tumor suppressing properties [11–15]. So far there is no report of redundancy for PTEN function, which could explain the high frequency with which PTEN inactivation is selected during tumor development [16]. By virtue of PTEN's ability to attenuate and control the extent of PI3K signaling, PTEN influences many cellular functions, including cell growth, survival, proliferation, and metabolism [8]. PTEN contributes to cell cycle regulation by blocking cells entering the S-phase of the cell cycle and by upregulation of p27kip1, which is recruited into the cyclin E/cdk2 complex [17], and downregulation of cyclin D1 [18]. Exogenous PTEN can suppress the growth of cells with mutated PTEN alleles [19], but the data of Tamura et al. [20] also indicates that this tumor suppressor has biological cell activity unrelated to growth. In contrast to many other tumor suppressors, which appear to have only nuclear roles, PTEN also appears to function in regulating dynamic cell surface interactions that involve integrins, FAK, cell migration, and the cytoskeleton [21–23].

In tumor tissue, proper function of PTEN acts as a tumor suppressor primarily through the ability to suppress proliferation and decrease cell survival. The frequent loss of PTEN function, through deletion, mutations, and/or decreased expression, is observed in hereditary cancers as well as sporadic cancers [8]. In many sporadic cancers, including breast cancer, PTEN is commonly found mutated at one allele. These monoallelic mutations of PTEN have been suggested to be as prevalent as p53 mutations found in most cancers and support the belief that PTEN is a bona fide tumor suppressor capable of controlling tumor initiation and progression. Germline mutations of PTEN are evident in hereditary autosomal dominant cancer syndromes, which have been collectively termed PTEN hamartoma tumor syndromes (PHTSs) [24, 25]. Many of these syndromes show increased risk of cancer occurring in the breast, thyroid, and endometrial tissues. It becomes apparent that some tissues are more prone to tumor initiation and progression in the absence of one PTEN allele, while other tissues often require both alleles deleted.

**Figure 1:**
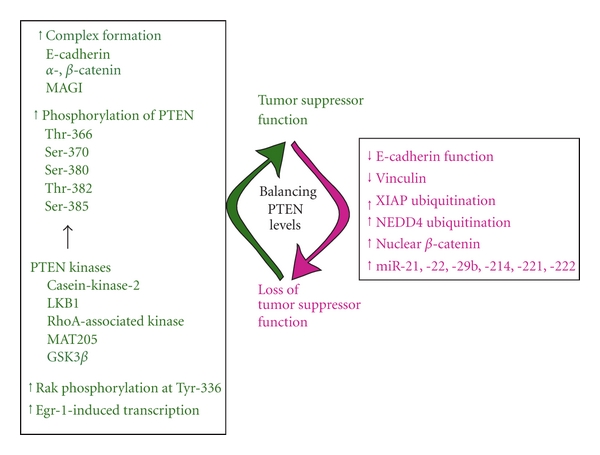
Mechanisms other than gene mutations and epigenetic silencing that regulate PTEN levels and ultimately its tumor suppressor function.

In the absence of germline and monoallelic mutations, PTEN protein levels have been found to be progressively lost during cancer progression [26]. A number of mechanisms, other than gene mutation and deletion, contribute to the loss or the decrease of PTEN protein levels in human cancers [27–31]. Proposed mechanisms for progressive loss of PTEN expression, in the absence of mutations, have been attributed to epigenetic events such as promoter methylation. Moreover, a large number of studies have indicated that posttranslational modifications on PTEN effects the protein's function, that is, phosphorylation and ubiquitination decrease PTEN protein levels, while oxidation and acetylation reduce PTEN activity [32]. Other reports suggest that E-cadherin expression or function may be an initiating cause of loss of PTEN expression in cancers, such as those that frequently occur in breast cancer, where PTEN expression is lost without identifiable mutations in the PTEN gene itself. Recently, several reports have indicated that PTEN shuttles between the nuclear and cytoplasmic compartments and that PTEN nuclear pool may have function to maintain genome integrity and cell-cycle control. Furthermore, the emerging field of microRNAs has revealed that PTEN is a direct and indirect target of some of these noncoding RNAs. Here we discuss the regulation of PTEN function by mechanisms other than gene mutations and epigenetic events as they relate to cellular function and oncogenic transformation (Figure 1).

## 2. Regulation of PTEN Protein Levels

The relevance of downregulated PTEN protein levels, as opposed to a complete loss of PTEN, is best observed in mouse models where PTEN is genetically manipulated [33–37]. In mice heterozygous for PTEN, where PTEN levels are essentially “50%” less when compared to wild type mice, a prevalence of tumors in the endometrium, thyroid, prostate, and liver as well as lymphomas was found. In contrast, mice with homozygous deletion of PTEN exhibited embryonic lethality by day 9.5 and displayed defective chorioallantoic development. Heterozygous deletions of PTEN indicate that PTEN haploinsufficiency predisposes some epithelial cells to becoming cancerous and this may explain why a large percentage of human epithelial tumors do not show any signs of gene mutation or deletion and instead retain wild-type copies of PTEN. PTEN transgenic mice, that express a range of PTEN levels (hypomorphs) all lower than wild type levels, have provided strong evidence that tissue response to PTEN function is truly dose-dependent [35, 37].

In some cancers, like nonsmall cell lung carcinoma (NSCLC), where PTEN expression is reduced or lost in 55%–74% of patients, genetic alteration such as loss of heterozygosity and epigenetic silencing were not good predictors of PTEN protein levels [29, 30]. With NSCLC, PTEN down regulation via ubiquitin-mediated degradation appears to be the primary mechanism of loss of PTEN function. The E3-ubiquitin ligase NEDD4 (neural precursor cell-expressed, developmentally downregulated 4-1) is overexpressed in 80% of NSCLC compared to normal tissue and has been shown to promote PTEN protein ubiquitin-mediated degradation and control PTEN stability in mouse tissue [28]. In light of these findings, NEDD4 overexpression in NSCLC has been suggested to be the primary factor in driving PTEN levels down in a large proportion of these cancers. In breast cancer, where PTEN protein is reduced in as much as 50% of cases, genetic and epigenetic alterations of the PTEN locus are rare, again suggesting that PTEN downregulation is occurring via a posttranslational event [38–40]. Similar to NSCLC, NEDD4 has been implicated in regulating PTEN turnover in breast cancer, a process that is inhibited by RAK phosphorylation of PTEN at Tyr-336 [41]. Unlike the Rak phosphorylation site on PTEN (Tyr-336), which stabilizes PTEN protein, phosphorylation at Thr-366, Ser-370, Ser-380, Thr-382, and Ser-385 has been attributed to attenuating PTEN stability [42]. It should be noted that Rak, which exhibits LOH in approximately 30% of breast cancers [43], has been subsequently termed a bona-fide tumor suppressor gene in breast cancer [41].

NEDD4 ubiquitin-ligase activity, which downregulates PTEN protein levels, is enhanced by the small endosomal PY-motif containing membrane proteins Ndfip1 and Ndfip2 [44]. This suggests that in normal cells the PTEN axis is balanced by opposing actions of Rak-induced PTEN stability and Ndfip1/Ndfip2 enhanced NEDD4-directed PTEN degradation—a shift in favor of NEDD4 activity predisposes cells to oncogenic transformation. Although strong evidence points to NEDD4 as an important regulator of PTEN protein levels in a number of cancer cells (bladder, gastric, and colorectal) [45, 46], gene knockout studies have challenged this assumption since in mice lacking NEDD4, PTEN protein levels remain stable with no evidence of increased stability in multiple cell types tested [47, 48]. Moreover, evidence from both in vitro and in vivo studies indicates that PTEN ubiquitination is still evident even in the absence of NEDD4. Other E3 ligases such as the X-linked inhibitor of apoptosis protein (XIAP) may also contribute in regulating PTEN protein levels. XIAP knockdown studies lower PTEN ubiquitination and upregulate PTEN stability, and conversely, overexpression of XIAP leads to PTEN poly-ubiquitination and lowered PTEN protein levels [49]. XIAP is a member of the inhibitor of apoptosis (IAP) family of proteins, and aside from its ability to act as E3-ligase, it is also capable of suppressing caspase-dependent cell death and is often upregulated in cancers [50, 51]. The phosphorylation state of PTEN also contributes to the regulation of PTEN subcellular localization and protein levels. PTEN phosphorylation at serine 380 (Ser-380) and threonines 382/383 (Thr-382/383) within its C-terminal tail strongly influences PTEN protein stability and its localization to the cell membrane. Several kinases, including casein kinase-2, LKB1, RhoA-associated kinase, the microtubule associated kinase MAT205, and GSK3*β*, have been reported to phosphorylate PTEN [52]. In light of the multiple pathways that may regulate PTEN protein levels, it becomes increasingly evident that these mechanisms can directly affect tumor initiation and progression.

## 3. PTEN and E-cad/MAGI

Calcium-dependent homophilic binding of the adhesion protein E-cadherin is vital for the cell-cell interaction found in epithelial tissue, while loss of E-cadherin expression is associated with transformation and metastatic cancers [53–55]. It has been demonstrated that PTEN interacts with cell adhesion molecules such as E-cadherin and MAGI-2 protein to inhibit migration and proliferation [56–59]. Although generally considered a cell-adhesion molecule, E-cadherin is also part of an active signaling network affecting a variety of cellular processes such as invasion and proliferation [60–65]. For instance, suppression of E-cadherin expression has been linked to the downregulation of PTEN expression and subsequent activity [66], while we have shown that restoration of E-cadherin increased PTEN protein levels in E-cadherin null breast cancer cells [67]. A cross-talk exists between PTEN and E-cadherin since PTEN also stabilizes E-cadherin at the adherens junction [68].

MAGI (membrane-associated guanylate-kinase inverted) family members are multidomain scaffolding proteins with multiple sites for protein interaction. There are multiple splice variants of MAGI; MAGI-1a, MAGI-1b, MAGI-2, and MAGI-3, where each is expressed in a tissue-specific manner. MAGI proteins are part of a PDZ subfamily of proteins called MAGUK (membrane-associated guanylate kinases). Members of this family, including MAGI-2 and -3, were shown to directly bind PTEN in yeast two-hybrid screens [56, 69, 70]. Soon after, mutational analysis showed that phosphorylation at Thr-382/383 greatly increases affinity of PTEN for MAGI-2 [58]. Kotelevets et al. put forth the idea of a signalosome composed of the scaffolding protein MAGI, *α*- and *β*-catenin, PTEN, PI3K, and E-cadherin [71]. In this complex PTEN is believed to be stabilized and protected from degradation, thus increasing its presence in the cytosol without affecting mRNA levels [57, 59]. Indeed, the importance of the association of the E-cadherin via the signalosome to the cytoskeleton is underscored by the loss of vinculin, which reduced E-cadherin mediated cell-cell binding [57, 72] and subsequently decreased PTEN protein levels [57]. PTEN levels were restored in vin^−/−^ cells by the overexpression of MAGI-2 or by expressing an E-cadherin-*α*-catenin fusion protein. [57]. Our previous work has shown that PTEN is recruited to cell-cell interactions with E-cadherin, MAGI-2, and *β*-catenin in a nonmalignant mammary acini model in laminin-rich extracellular matrix. In this model, either inhibition of E-cadherin function or reduction of PTEN protein levels abrogated acini organization and proliferation control suggesting that PTEN roles in cell adhesion and proliferation may be interconnected [67]. The surprising sensitivity of mammary cells to the PTEN regulatory node may explain why it is so low in a variety of solid tumors where tissue structure is compromised. These findings suggest that PTEN-dependent changes in PI3K signaling may operate in conjunction with other E-cadherin-dependent processes to cause cessation of cell growth.

*β*-catenin is part of the cadherin-catenin complex and indirectly connects E-cadherin to the cytoskeleton. Studies have suggested that *β*-catenin negatively regulates PTEN transcription by blocking early growth response gene 1 (Egr1) [66]. Egr1 has been shown to activate PTEN transcription by directly binding to the Egr1 binding motif in the PTEN promoter [73]. Taken together, a picture arises depicting the relationship between E-cadherin, PTEN, MAGI, and loss of cell-cell adhesion in cancer. When present, E-cadherin stabilizes the adherens junction complex, including the signalosome, which sequesters *β*-catenin at the plasma membrane. Thus, *β*-catenin cannot translocate to the nucleus and inhibit Egr1 transcription, the effect of which is continued PTEN transcription. Additionally, PTEN is protected in the signalosome from proteosomal degradation, resulting in sustained PTEN protein levels in the cytosol and subsequently negative regulation of the PI3K/AKT pathway. Loss of E-cadherin expression in cancer causes disruption of the signalosome, thus freeing *β*-catenin and PTEN from the MAGI scaffolding protein. The net effect of this is the proteosomal degradation of PTEN and the inhibition of PTEN transcription by *β*-catenin that has translocated to the nucleus.

## 4. Nuclear PTEN

**Figure 2:**
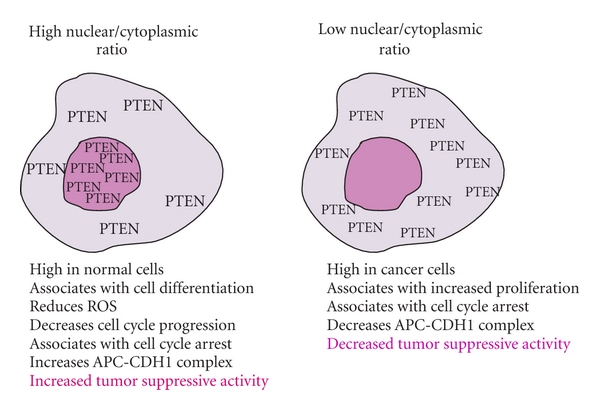
Cell-characteristics associated with the presence or absence of nuclear PTEN.

The existence of nuclear PTEN was reported in a number of cell models including primary neurons and endothelial cells [74], myoepithelial cells of normal breast ducts [75], and normal follicular thyroid cells [76]. In general stronger PTEN nuclear staining has been observed in normal cells when compared to tumor cells [52] and is thought to directly correlate with cell differentiation [77]. The presence of nuclear PTEN was at first thought to be an artifact based on immunohistochemical techniques; however, a proposed role for nuclear PTEN was provided from studies examining melanoma tumors where a decrease in PTEN nuclear levels correlated with an increase in the proliferation status of tumor cells [78]. As well, an investigation with colorectal adenocarcinoma showed a gradual decrease in nuclear PTEN expression from normal to subsequent progression stages of metastasis [79]. Subcellular fractionation and immunocytochemistry studies have confirmed that nuclear translocation of PTEN in the human breast carcinoma cell line MCF7 was cell cycle dependant [80]. This cell line has also demonstrated that a rise in nuclear PTEN level occurs at G0-G1 with a decrease in nuclear PTEN level in S-phase, indicating nuclear PTEN to be involved as a cell cycle modulator [81]. Further studies using the MCF7 cell line found that nuclear PTEN is required for cell cycle arrest and cytoplasmic PTEN is required for apoptosis [81]. In contrast to breast cancer cells, increased level of nuclear PTEN was associated with G2 arrest in melanoma cells instead of G1 [82]. These findings indicate that nuclear localization of PTEN is associated with decreased proliferation. These studies and others indicated that increased nuclear translocation of PTEN is associated with PTEN tumor-suppressing activity and nuclear PTEN depletion correlates with increased tumor progression [76, 78, 83, 84] (Figure 2).

The absence of a “true” nuclear localization signal within PTEN has led to a number of alternative mechanisms for nuclear entry of PTEN [85]. One mechanism may be simple diffusion, which was found with a GFP-PTEN fusion protein construct entering the nucleus of HeLa cells [86]. It should be noted that proteins with molecular mass greater than 60 kDa are generally prohibited from nuclear entry by diffusion [87, 88]. However, diffusion does not explain the differential distribution pattern of PTEN in differentiated/nondividing resting cells and tumor cells. Therefore, it has been suggested that an active transport system for PTEN nuclear trafficking must exist. One such mechanism may involve the small GTPase RAN, which is a well-studied regulator of importin-mediated nuclear transport [89]. Upon investigating this mechanism, it was found that a GTPase deficient dominant negative RANQ69L mutant excluded nuclear entry of PTEN [90]. It should be noted that this RAN-dependent mechanism of PTEN nuclear import is also associated with increased cellular apoptosis in the human glioblastoma cell line U87MG. Another mechanism of active nuclear import of PTEN may involve the Major Vault Protein (MVP), which is a carrier molecule involved in nuclear-cytoplasmic transport of molecules [91]. Evidence was provided that PTEN binding to MVP promoted nuclear translocation of PTEN in yeast-two hybrid system [92]. Later it was confirmed in 293T and HeLa cell lines that it was a Ca^+2^-regulated interaction and the binding was specific between the C2 PTEN domain and the calcium binding motif (EF-hand pair) of MVP [85]. Other mechanisms of PTEN nuclear import may involve posttranslational modifications of PTEN. For instance, polyubiquitination of PTEN results in PTEN degradation, whereas monoubiquitinated PTEN at K289 undergoes nuclear translocation [45]. Phosphorylation of PTEN is also another posttranslational modification of PTEN that has been shown to directly affect nuclear translocation. The C-terminal domain of PTEN possesses important phosphorylation sites (Ser-380, Thr-382/383, Ser-385) which are involved in regulation of PTEN stability, activity, and localization [42, 93–98]. Phosphorylation of PTEN at Ser-380 is upregulated during oxidative stress and associates with an accumulation of nuclear PTEN [98]. The outcome of this accumulation of nuclear PTEN leads to growth arrest, cellular apoptosis, and a reduction of reactive oxygen species production [98]. Findings have also indicated that nuclear localization of PTEN is regulated by the PI3K pathway. Specifically, cells with dominant negative Akt mutants or those treated with inhibitors of PI3K mTOR suppress PTEN nuclear transport, while siRNA silencing for S6K 1/2 (protein kinase) also blocks PTEN nuclear entry. Together these data suggest that PI3K/Akt/mTOR/S6K activation leads to PTEN nuclear transport [99]. Finally, it has been reported that LKB1/CaMKK-(Ca^+2^/calmodulin-dependant protein kinase) mediated activation of AMPK *α*1/2 can bypass the inhibition of PTEN nuclear entry achieved with mTOR/S6K downregulation [100] and thus provides another mechanism for PTEN entering the nucleus. 

In seminal work, the functional significance of nuclear PTEN with regard to proliferation and cell cycle control has been revealed [101]. In this particular study, it was found that PTEN nuclear exclusion rather than its phosphatase inactivation was responsible for decreased activation of the APC-(Anaphase-promoting complex/cyclosome-) CDH1 tumor suppressor complex. Essentially, nuclear PTEN increases the activity of APC/C complex and induces its association with CDH1. APC/C imparts a major regulatory control on the cell cycle from mitosis to late G1 phase [102, 103], and its functional activity is maintained through its interaction with CDC20 and CDH1, while CDH1 activity is restricted between late mitosis and G1 [104, 105]. The APC-CDH1 complex is essential for cell-cycle regulatory control and tumor-suppressive activity [101]. By virtue of regulating the APC/CDH1 complex nuclear PTEN becomes an important factor in determining cellular outcome and proliferative status. This is an important finding related to cancer therapy since, it has been also shown that loss of PTEN sensitizes cells to pharmacological inhibition of PLK1 and Aurora kinases.

## 5. Regulation of PTEN by MicroRNAs

MicroRNAs (miRNAs) are short 18–25 nucleotide long noncoding RNAs involved in posttranscriptional regulation of gene expression [106]. They negatively regulate target gene expression through complimentary binding to the 3′ untranslated region (UTR) of mRNAs [107–111]. MicroRNAs have gained importance in development and progression of several types of cancer that can be explained by their ability to modulate the expression of gene products involved in cell migration, invasion, and apoptosis [112, 113]. Overexpression of certain microRNAs like miR-21, miR-10b, miR-373, and miR-155 has shown to promote metastasis in cancer whereas downregulation of other miRNAs such as miR-31, let-7, miR-146, and miR-193b has been shown to promote tumor growth, metastasis, and invasion [114–117].

In 2007, Meng et al. reported overexpression of miR-21 in human hepatocellular cancer using miRNA microarray assay [27]. Inhibition of miR-21 was accompanied by an increase in PTEN expression with a decrease in cell migration, invasion and cell proliferation. Downmodulation of miR-21 also affected the downstream effectors of PTEN such as FAK phosphorylation as well as MMP-2/9 expression involved in cell migration and invasion. This original work supported that PTEN was a direct target of miR-21. Later miR-21 was also reported to be overexpressed in nonsmall cell lung cancer cells (NSCLCs) compared to adjacent nontumor cells with an inverse corelation to PTEN expression [118]. NSCLC cell lines transfected with miR-21 inhibitor resulted in increased luciferase reporter activity for PTEN 3′ UTR, suggesting that miR-21 binds to the 3′ UTR of PTEN mRNA. Additionally, an elevation in PTEN protein level was detected without any effect on PTEN mRNA levels. This work supported the hypothesis that miR-21 down regulates posttranscriptional expression of PTEN. Further support for the role of miR-21 was provided by an *in vivo* mouse model in which miR-21 was knocked out. This increased the expression of several miR-21 target genes including PTEN [119]. In contrast to these studies, upregulation of miR-21 in normal and tumor breast cells did not associate with a detectable change in PTEN levels as determined by miRNA in situ hybridization techniques [120]. Recent work has demonstrated that the oncogenicity of miR-21 oncomir (oncogenic miRNA) could be cell and tissue dependant and must be contextualized to a particular disease prior to consideration as a therapeutic target [121].

MicroRNAs other than miR-21 have been shown to be involved in various biological events and disease through PTEN regulation. For instance, MiR-214 is involved in cell survival [122] and cell apoptosis [123] and has been found to be overexpressed in gastric cancer cells compared to normal gastric mucosal cell lines using real-time PCR techniques [124]. Transient transfection with antisense miRNA-214 oligonucleotides downregulated miR-214 expression with a significant increase in PTEN expression. Flow cytometry revealed G1 increases and S-phase decreases in gastric cancer cells when miRNA-214 was downregulated. PTEN was also reported to be the direct target of miR-29b in human breast cancer cells [125]. The breast cancer cell line MDA-MB-231, which migrates and invades faster than MCF7 cells, was found to display higher miR-29b levels compared to MCF7 cells. Inhibition of miR-29b in MDA-MB-231 cells increased PTEN expression, which promoted apoptosis and reduced cell migration and invasion. It was also reported that knockdown of miR-221 and miR-222 in multiple cancer cell lines upregulated PTEN expression suppressed of AKT activity with enhanced radio sensitivity in tumor cells [126]. The previous findings have indicated that PTEN is a target of a number of microRNAs and that cancer cells upregulate these regulatory RNAs to downregulate the tumor suppressor characteristics of PTEN.

## 6. Conclusions

There is ample evidence that the full functionality of PTEN is modulated by alternative mechanisms beyond gene mutations and epigenetic processes. For instance a number of tissue-specific cancers strongly associate with PTEN deregulation at the gene expression level, changes in PTEN posttranslational modifications, miss-guided PTEN subcellular localization, and PTEN-specific microRNA upregulation. Determining whether these alternative mechanisms are causal or a consequence of tumor initiation and progression or whether they produce tissue-specific effects is still an ongoing area of important research. Clinically, as PTEN-regulating pathways become fully resolved, essential factors will arise, hopefully providing new targets for the development of novel and effective anticancer therapies and diagnostic tools.
